# Supplementary material for: The interactome of intact mitochondria by cross-linking mass spectrometry provides evidence for coexisting respiratory supercomplexes
Source: Mol Cell Proteomics. 2017 Dec 8;17(2):216–32. doi: 10.1074/mcp.RA117.000470 (PMC5795388; doi:10.1074/mcp.RA117.000470)
Supplement: Supplemental Data [file supp_RA117.000470_133922_0_supp_23978_fzffwf.zip › spectra_annotation/mito_DR_spectra_annotation/495-1-13-1-4-1.pdf]

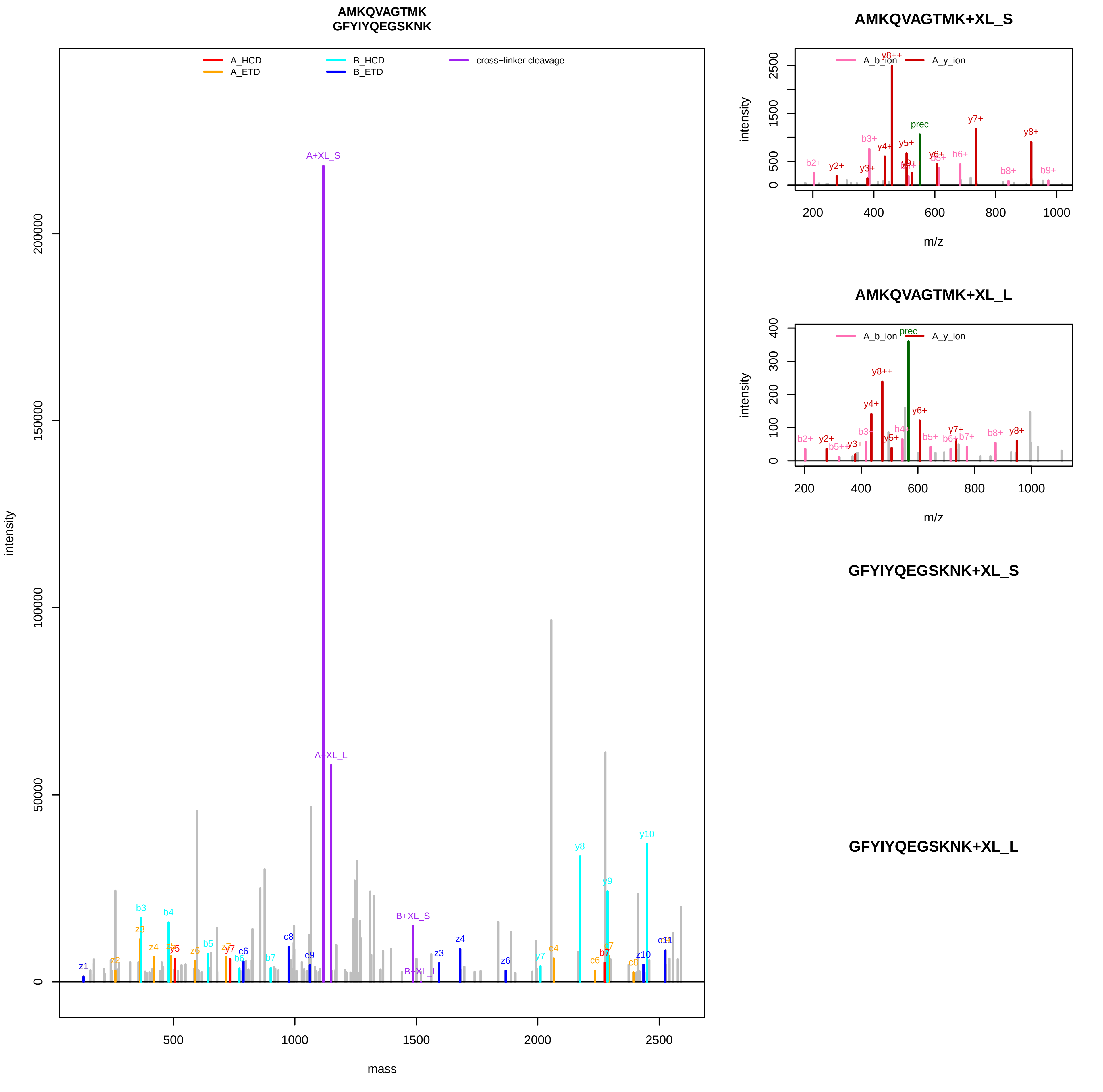
AMKQVAGTMK
GFYIYQEGSKNK
A_HCD
A_ETD
B_HCD
B_ETD
cross−linker cleavage
A+XL_S
A+XL_L
B+XL_S
B+XL_L
intensity
mass
0
50000
100000
150000
200000
500
1000
1500
2000
2500
z1 z2 b3 z3 z4 b4 z5 y5 z6 b5 z7 y7 c6 b6 b7 c8 c9 z3 z4 z6 y7 c4 y8 c6 b7 y9 c7 c8 z10 y10 c11
AMKQVAGTMK+XL_S
A_b_ion
A_y_ion
intensity
m/z
prec
b2+ y2+ y3+ b3+ y4+ y8++ y5+ y9++ y6+ b5+ b6+ y7+ b8+ y8+ b9+
AMKQVAGTMK+XL_L
b2+ y2+ b5++ y3+ b3+ y4+ y8++ y5+ b4+ prec y6+ b5+ b6+ y7+ b7+ b8+ y8+
GFYIYQEGSKNK+XL_S
GFYIYQEGSKNK+XL_L
